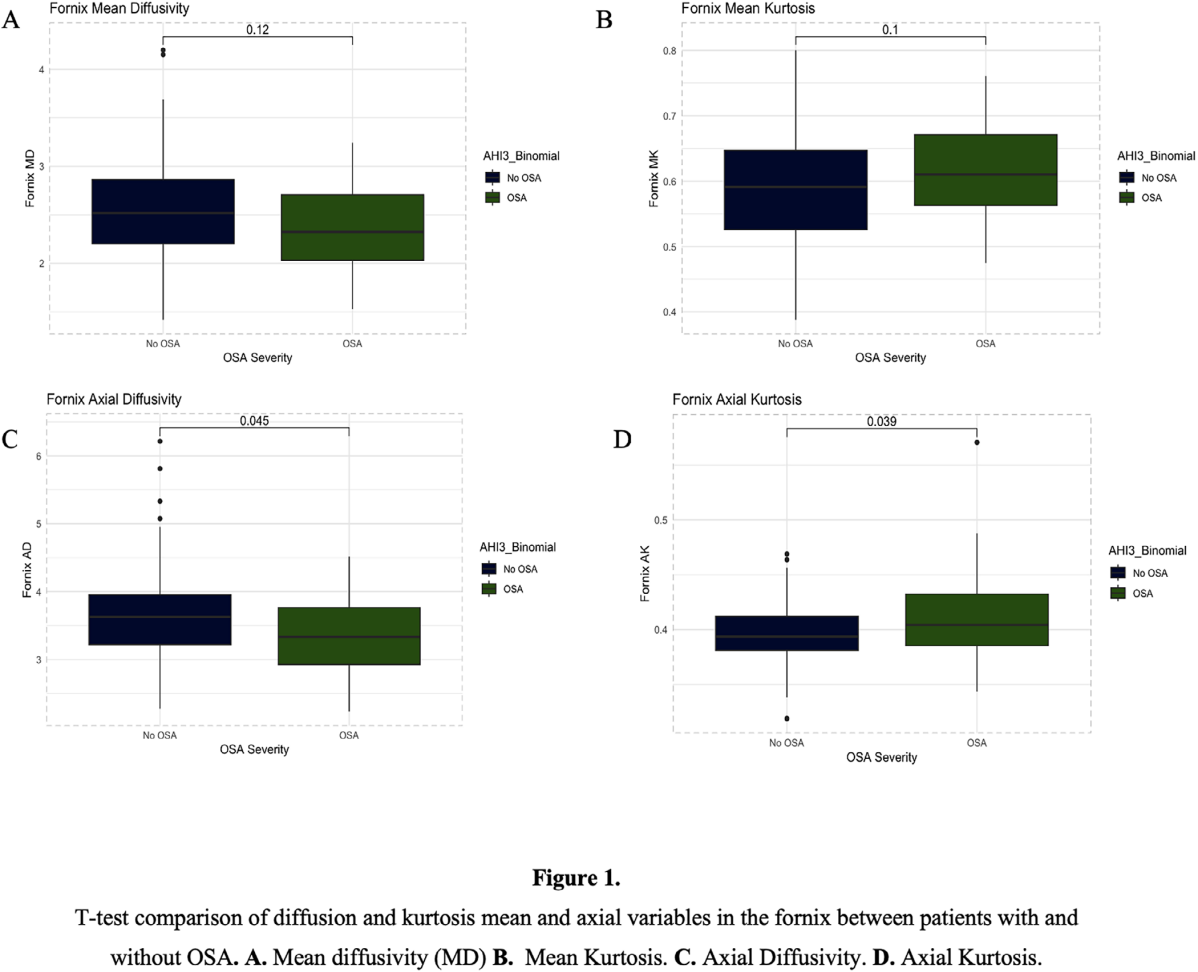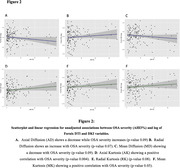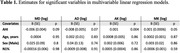# DTI and DKI differences in the fornix microstructure associated with sleep apnea in cognitively normal patients

**DOI:** 10.1002/alz.087616

**Published:** 2025-01-09

**Authors:** Luisa F Figueredo, Jenny Chen, Ankit Parekh, Omonigho M Bubu, Els Fieremans, Ricardo Osorio

**Affiliations:** ^1^ NYU Grossman School of Medicine, New York, NY USA; ^2^ New York University Grossman School of Medicine, New York, NY USA; ^3^ Mount Sinai School of Medicine, New York, NY USA

## Abstract

**Background:**

Obstructive sleep apnea (OSA) is a complex and heterogeneous condition associated with chronic physiological and neuropsychological disturbances (1–4). One notable neuropsychological effect observed in OSA patients is memory impairment (2,5). Additionally, some reports suggest that OSA may be associated with Alzheimer’s Disease (AD) (4). Memory consolidation is regulated through a cohesive network of structures, including the hippocampus, fornix, anterior thalamus, and mammillary bodies (6). Diminished mammillary body and hippocampal volume have been described in OSA patients compared to normal subjects, suggesting potential circuit changes (2). This study aims to comprehensively evaluate the effects of OSA on the fornix microstructure in cognitively normal patients, measured by DTI and DKI variables.

**Methods:**

One‐night polysomnography, neurocognitive assessment, and brain diffusion MRI (dMRI) with DTI (Diffusion Tensor Imaging)/DKI (Diffusion Kurtosis Imaging) protocol were performed in 145 individuals with a mean age of 65 [61‐70] and a mean Mini‐Mental‐Score (MMSE) of 29 [28‐30]. OSA measurements included the Apnea‐Hypopnea Index (AHI) and variables related to sleep duration and fragmentation. Microstructural properties of white matter tracts (DTI. DKI (8–10)) were estimated after preprocessing dMRI using DESIGNER pipeline (https://arxiv.org/abs/2305.14445).

**Results:**

A correlation matrix between AHI3% and AHI4% revealed positive correlations with Axial Kurtosis (AK) and axial diffusion (ADi), and negative correlations with Mean Diffusivity (MD) and Mean Kurtosis (MK). Figure 1C‐1D illustrates a significant difference between patients with and without OSA. Associations between AHI3% and AHI4% and log Fornix FA, MD, RD, ADi, MK, RK, AK, De″, De⊥ and Da were tested while adjusting for age, sex, hypertension, APOe4, BMI, and N1%, N3%. Unadjusted linear regression models appear in Figure 2, where only AK (Figure 2D), and MK (Figure 2F) showed statistical significance. Finally, multivariable‐adjusted models for AHI3% revealed a significant association with AK and MD only (Table 1).

**Conclusion:**

OSA may be associated with microstructural changes in the fornix in cognitively normal patients, particularly in those with severe OSA. This study might contribute as a baseline for future works that include progression to AD.